# Ovulation induction or artificial programmed protocol: which endometrial preparation approach is more favorable in reducing obstetrical and neonatal complications for ovulation disorder patients undergoing frozen-thawed embryo transfer?

**DOI:** 10.3389/fendo.2026.1784899

**Published:** 2026-04-07

**Authors:** Shuxia Ma, Zhiqin Bu, Jie Lu, Keyan Wang

**Affiliations:** 1 Reproductive Medicine Center, Luoyang Maternal and Child Health Hospital, Luoyang, China; 2 Reproductive Medicine Center, Shanghai Sixth People’s Hospital Affiliated to Shanghai Jiao Tong University School of Medicine, Shanghai, China; 3 Center for Reproductive Medicine, Henan Key Laboratory of Reproduction and Genetics, The First Affiliated Hospital of Zhengzhou University, Zhengzhou, China; 4 Henan Institute of Medical & Pharmaceutical Sciences, Zhengzhou University, Zhengzhou, China

**Keywords:** estrogen-progesterone cycle, frozen-thawed embryo transfer, obstetrical and neonatal complications, ovulation induction, pregnancy outcomes

## Abstract

**Objective:**

Several endometrial preparation protocols are available in frozen-thawed embryo (FET) transfer cycles. The aim of this study was to compare the clinical outcomes, obstetrical and neonatal complications between ovulation disorder patients undergoing FET with ovulation induction protocol and patients with artificial programmed protocol.

**Methods:**

A single-center retrospective cohort study using propensity-score matching (PSM) was conducted. Ovulation disorder women undergoing FET using ovulation induction medicine Letrozole (OI group), or estrogen/progesterone (EP) for endometrial preparation were included. Clinical outcomes, obstetrical and neonatal complications were compared before and after PSM. Multiple logistic regression models were performed to demonstrate the independent impact of endometrial protocols on pregnancy outcomes.

**Results:**

A total of 552 women in OI group and 3344 women in EP group were included. As for pregnancy outcomes after PSM, patients in OI group were with significantly higher live birth rate (38.1% vs. 33.4%; P = 0.042), and lower early spontaneous miscarriage rate (13.1% vs. 18.9%; P = 0.032). In singleton birth, the prevalence of GDM (7.9% vs. 14.6%; P = 0.037), and LBW (7.9% vs. 14.1%; P = 0.042) was significantly lower in patients from OI group as compared with EP group. In patients after PSM, after adjusting for covariates, EP protocol was a risk factor of early spontaneous miscarriage rate (OR = 1.450, 95%CI: 1.085-1.963; P = 0.029), GDM (OR = 1.627, 95%CI: 1.288-2.601; P = 0.034), and LBW (OR = 1.852, 95%CI: 1.127-2.250; P = 0.041).

**Conclusion:**

As compared with EP protocol, using Letrozole to achieve endometrial preparation prior to FET may yield a decreased risk of early spontaneous miscarriage rate, GDM, and as well as LBW in ovulation disorder patients undergoing FET.

## Introduction

Nowadays, frozen-thawed embryo transfer (FET) has been commonly used in fertility centers aiming to improve cumulative pregnancy rates. As compared with fresh embryo transfer cycles, the procedure of FET is simple and much easier to operate; however, it is also important to synchronize the endometrium with the developmental stage of the embryo to facilitate implantation (1). Thus, endometrial preparation is critical for the success of embryo implantation in FET cycles. Currently, the three most commonly used methods for preparing the endometrium in FET cycles are artificial programmed protocol (Estrogen-progesterone, EP), natural cycle (NC), and ovulation induction (OI) protocol (2, 3). The major difference among these three protocols is that in the EP protocol, there is no ovulation and therefore no corpus luteum formation during pregnancy.

To date, several studies have demonstrated that, as compared with EP protocol, NC was found to be more favorable in reducing obstetrical and neonatal complications after FET in ovulatory women (4, 5), which is consistent with results from a recent Meta-analysis by Zaat et al, which showed that birthweight was lower following NC protocol versus EP protocol. In addition, NC compared to EP resulted in a lower risk of large for gestational age, macrosomia, low birthweight, preterm birth, very preterm birth, hypertensive disorders of pregnancy, pre-eclampsia, placenta previa, and postpartum hemorrhage (6). It is recommended that for patients with regular ovulation, the NC protocol is more suitable, implying that the importance of corpus luteum during pregnancy (7).

For patients with regular ovulation, it seems reasonable that NC protocol should be the preferred choice. However, for patients with ovulatory disorders, NC protocol is not suitable; therefore, there are EP and OI protocols available for them to choose. In 2017, Tatsumi et al. found that OI protocol using Letrozole may improve clinical pregnancy, live births and reduce the risk of miscarriage in patients undergoing single FET cycles. However, neonatal outcomes were mostly similar in OI, EP, and NC groups (8).In contrast with results from this study, two more recent studies in polycystic ovary syndrome (PCOS) women compared the efficacy and safety between OI protocol and artificial programmed protocol (9, 10), both indicating that OI protocol was associated with a lower risk of hypertensive disorder of pregnancy. It seems that for patients with PCOS, using the OI protocol for endometrial preparation is worthwhile, even though the EP protocol is simpler.

Thus, the aim of this study was to compare the clinical outcomes, obstetrical and neonatal compilations in ovulation disorder patients undergoing FET with OI and EP protocol, using propensity score matching (PSM) method to control possible biases.

## Materials and methods

### Study population

This study was approved by the Medical Ethics Committee of Luoyang Women and Child Health Hospital (SZSL2024122501) on 25th, December, 2024. All data were extracted from electronic database without any personally identifiable information, and the requirement for informed consent was waived since the retrospective nature of this study.

This retrospective study included patients who visited the Reproductive Medicine Center of Luoyang Women and Child Health Hospital from January 2015 to December 2022 undergoing FET. The main inclusion criteria included: (1) first FET cycle; (2) ovulation disorder women, which mainly include PCOS, dismissed ovarian reserve, hypothalamic dysfunction, excess prolactin, etc.; (3) using ovulation induction medicine Letrozole, or estrogen/progesterone for endometrial preparation. Exclusion criteria were: (1) uterine malformations, intrauterine adhesions and polyps; uterine fibroids and adenomyosis; (2) sperm/oocyte donation cycles; (3) vanishing twin, or multifetal pregnancy reduction cycles; (5) history of recurrent spontaneous miscarriage, pre-existing diabetes and hypertension before pregnancy.

### Endometrial preparation protocols

Briefly, in OI protocol, Letrozole was given orally for 5 days, beginning on day 5 of the menstrual cycle (5 mg twice daily). The diameter of the dominant follicle was examined 5 days later. When the diameter of the dominant follicle reached 14mm, a blood sample was obtained for progesterone and LH levels. If follicle diameters were >18 mm, endometrial thicknesses >8 mm, patients were given 10,000 U human chorionic gonadotropin (HCG, Livzon Pharmaceutical; Zhuhai, China) to induce ovulation based on doctor’s experience.

For EP cycles, patients began oral estradiol (4 mg; Progynova; Bayer, Leverkusen, Germany) twice a day on cycle day 3. This dosage was adjusted based on endometrial thickness every 7 days. After 14–18 days, an ultrasound was performed. If no leading follicle was present, endometrial thickness> 8mm, blood progesterone < 3 ng/mL, progesterone in oil was added for endometrial transformation.

Luteal phase support after embryo transfer was vaginal exogenous progesterone supplementation (400 mg/d; Utrogestan; BesinsHealthcare), and oral dydrogesterone (20 mg; Duphaston; Solvay Pharmaceuticals B.V., Veenendaal, The Netherlands). After pregnancy, luteal phase support dosage was adjusted according to serum progesterone levels, and treatment was administered until 2 months after embryo transfer.

### Main outcome measures

Implantation rate was defined as the proportion of fetal hearts relative to the number of embryos transferred. Positive pregnancy test was defined as a serum hCG>50mIU/mL 2 weeks after embryo transfer. Clinical pregnancy was the actual pregnancy confirmed by ultrasound by transvaginal transducer with the presence of amniotic sac, embryo, fetal heart after 4–5 weeks of embryo transfer. Early spontaneous miscarriage was defined as loss of clinical pregnancy that occurred before 12 weeks of gestation. Live birth was defined as the delivery of a viable infant at≥ 28 gestational weeks.

Hypertensive disorders of pregnancy (HDP) was defined as the development of blood pressure > 140/90 mm Hg after pregnancy with or without protein uria or other signs of preeclampsia, including preeclampsia and gestational hypertension and excludes chronic hypertension. Gestational diabetes mellitus (GDM) was defined as any degree of glucose intolerance with onset or first recognition during pregnancy. Preterm premature rupture of membranes (PPROM) was defined as rupture of membranes before37 weeks of gestation. Preterm birth (PTB, 32–37 weeks), very PTB (28–32 weeks), low birthweight (LBW, 1500–2500 g), very LBW (<1500g), congenital malformation was defined as previous literature (11). Data of obstetrical and neonatal outcomes were from electronic medical record system in Luoyang Maternal and Child Health Hospital, or from Hospital Discharge Record in other hospitals and reviewed by our trained nurses.

### Statistical analysis

The basic characteristics and pregnancy outcomes of patients included were analyzed according to the endometrial preparation protocols used (OI, and EP). Given that baseline parameters were significantly different between OI group and EP group, PSM was performed to control biases between these two groups. The propensity score was calculated by using a multiple logistic regression model, with OI versus EP serving as the dependent variable, and female age, body mass index (BMI), serum anti-Mullerian hormone (AMH), infertility diagnosis, number and type of embryos transferred serving as independent variables. To optimize the precision of the study, PSM was conducted with a caliper width of 0.2 of the SD of the logit of the propensity score. The ratio of matching was 1:4 by closest neighbor matching (12).

Firstly, basic parameters, pregnancy outcomes, obstetrical and neonatal complications were compared in OI and EP groups before and after PSM. Then, multivariate logistic regression analysis was also performed to explore risk factors for live birth, early miscarriage, GDM, LBW, and PTB. Differences between these two groups were detected by Student’s t-tests or Chi-square test. Statistical analysis was performed with SPSS (Statistical Package for Social Science, SPSS Inc, Chicago, IL, USA) version 23.0. A two-tailed P< 0.05 was considered statistically significant.

## Results

**Figure 1:**
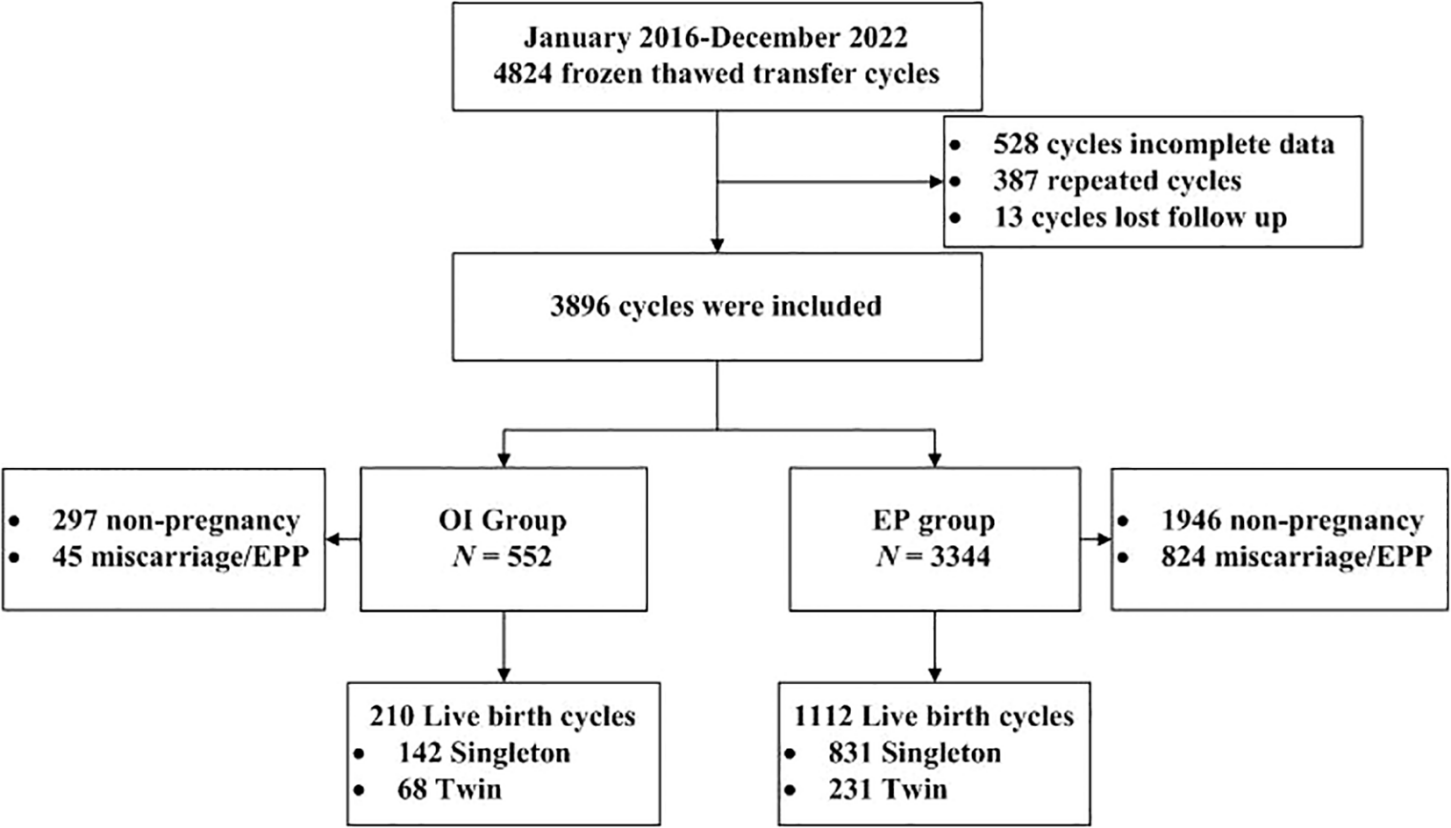
Flowchart of the retrospective cohort study. OI, ovulation induction; EP, estrogen-progesterone; EPP, ectopic pregnancy.

A total of 4824 ovulation disorder patients undergoing FET treatment cycles were included. After firstly screening for repeated cycles, missing data cycles, and lost follow up cycles, the final number of OI and EP cycles was 552 and 3344, respectively. A brief flow chart of patient selection was shown in **Figure 1**.

**Table 1** presented patient baseline parameters between these two groups. Before PSM, patients in OI group were younger than those in EP group (32.2 vs. 33.2 years; P< 0.001). In addition, the composition of infertility type, and infertility diagnosis were also significantly different between groups. More percentage of women was primary infertility (22.6% vs.16.9%; P< 0.001), and was diagnosed with PCOS (60.9% vs. 46.9%; P< 0.001) in OI group. In OI group 71.7% of patients were transferred with blastocyst, and the average number of embryos transferred was 1.8, which was significantly lower than that in EP patients (1.8 vs. 1.6; P< 0.001). However, after PSM, all these baseline parameters were comparable between groups. As for pregnancy outcomes before PSM, implantation rate (34.4% vs. 31.1%; P = 0.035), and live birth rate (38.0% vs. 33.3%; P = 0.028) were also higher in patients from OI group; while early spontaneous miscarriage rate was lower (13.3% vs. 18.6%; P = 0.043). Moreover, after PSM, patients in OI group were also with significantly higher live birth rate (38.1% vs. 33.4%; P = 0.042), and lower early spontaneous miscarriage rate (13.1% vs. 18.9%; P = 0.032).

**Table 1:** Basic characteristics and clinical outcomes before and after propensity-score matching (PSM).

Characteristics	Before PSM			After PSM		
	OI	EP	*P*	OI	EP	*P*
No. of cycles	552	3344		536	2144	
Female age (y)	32.2 ± 5.0	33.2 ± 5.1	< 0.001	32.2 ± 4.9	32.3 ± 5.0	0.122
BMI (Kg/m^2^)	22.1 ± 2.6	22.9 ± 2.8	0.026	22.1 ± 2.6	22.2 ± 2.6	0.475
Infertility duration (y)	3.1 ± 2.3	3.9 ± 2.5	< 0.001	3.2± 2.3	3.3± 2.4	0.480
Basal FSH (mIU/mL)	5.6 ± 1.2	6.2 ± 1.5	< 0.001	5.6 ± 1.2	5.7 ± 1.3	0.286
AMH (ng/mL)	3.3 ± 2.1	2.4 ± 2.0	< 0.001	3.2 ± 2.0	3.1± 2.0	0.146
Infertility type, %			< 0.001			0.720
Primary infertility	22.6 (125/552)	16.9 (566/3344)		21.1 (113/536)	20.4 (437/2144)	
Secondary infertility	77.4 (427/552)	83.1 (2778/3344)		78.9 (423/536)	79.6 (1707/2144)	
Infertility diagnosis, %			< 0.001			0.185
PCOS	60.9 (336/552)	46.9 (1568/3344)		60.6 (325/536)	59.2 (1269/2144)	
Dismissed ovarian reserve	20.7 (114/552)	36.2 (1210/3344)		20.9 (112/536)	22.1 (475/2144)	
Others^*^	18.5 (102/552)	16.9 (566/3344)		18.5 (99/536)	18.7 (400/2144)	
No. of embryos transferred	1.8 ± 0.3	1.6 ± 0.4	< 0.001	1.7 ± 0.3	1.7 ± 0.3	0.120
Stage of embryos transferred, %			< 0.001			0.113
Cleavage	28.3 (156/552)	15.2 (508/3344)		26.5 (142/536)	23.2 (498/2144)	
Blastocyst	71.7 (396/552)	84.8 (2836/3344)		73.5 (394/536)	76.8 (1646/2144)	
Endometrial thickness (mm)	10.5 ± 2.8	10.8 ± 2.7	0.018	10.6 ± 2.8	10.6 ± 2.7	0.146
Implantation rate, %	34.4 (354/1028)	31.1 (1702/5471)	0.035	33.7 (308/914)	32.9 (1202/3648)	0.667
Positive pregnancy test rate, %	49.6 (274/552)	45.1 (1507/3344)	0.046	48.1 (258/536)	46.1 (988/2144)	0.394
Clinical pregnancy rate, %	46.2 (255/552)	41.8 (1398/3344)	0.053	45.7 (245/536)	44.3 (950/2144)	0.560
Early spontaneous miscarriage rate, %	13.3 (34/255)	18.6 (260/1398)	0.043	13.1 (32/245)	18.9 (180/950)	0.032
Live birth rate, %	38.0 (210/552)	33.3 (1112/3344)	0.028	38.1 (204/536)	33.4 (716/2144)	0.042
No. of newborns			0.032			0.712
Singleton	67.6 (142//210)	74.7 (831/1112)		68.6 (140/204)	70.0 (501/716)	
Twin	32.4 (68/210)	25.3 (281/1112)		31.4 (64/204)	30.0 (215/716)	

OI, ovulation induction; EP, estrogen-progesterone; BMI, body mass index; FSH, follicle stimulation hormone; AMH anti-Müllerian hormone; PCOS, polycystic ovarian syndrome. Data were presented as means ± SD, % (n/N). Differences were analyzed by Student’s t-test, or chi-squared test.^*^ Others mainly include hypothalamic dysfunction, excess prolactin, ect.

As shown in **Table 2**, before PSM, for patients with singleton birth, OI patients were with lower prevalence of GDM (7.7% vs. 15.4%; P = 0.016), PTB (5.6% vs. 11.4%; P = 0.038), and LBW (7.7% vs. 16.0%; P = 0.010). However, other complications such as HPD, PPROM, VPTB, and VLBW were comparable between two groups. In addition, for patients with twin birth, except for prevalence of LBW, which was significantly lower in OI patients as compared with that in EP patients (44.1% vs. 59.8%; P = 0.019), other obstetrical and neonatal complications were all comparable.

**Table 2:** Obstetrics and perinatal outcomes of live births in OI and EP groups before propensity-score matching (PSM).

	Singleton			Twin		
	OI	EP	*P*	OI	EP	*P*
Obstetrics outcomes	142	831		68	281	
HDP	3.5 (5/142)	6.0 (50/831)	0.234	7.4 (5/68)	7.8 (22/281)	0.895
GDM	7.7 (11/142)	15.4 (128/831)	0.016	11.8 (8/68)	17.1 (48/281)	0.284
Placenta previa	5.6 (8/142)	6.6 (55/831)	0.679	5.9 (4/68)	10.0 (28/281)	0.659
PPROM	2.8 (4/142)	6.1 (51/831)	0.165	4.4 (3/68)	9.3 (26/281)	0.230
PH	5.6 (8/142)	8.4 (70/831)	0.258	8.8 (6/68)	12.5 (35/281)	0.404
Caesarean section	71.8 (102/142)	78.5 (652/831)	0.080	85.3 (58/68)	87.2 (245/281)	0.671
Perinatal outcomes						
Average gestational age (week)	38.9 ± 2.4	38.0± 2.3	0.025	35.9 ± 1.8	35.4 ± 1.7	0.188
PTB (32–37 weeks)	5.6 (8/142)	11.4 (95/831)	0.038	44.1 (30/68)	55.2 (155/281)	0.102
VPTB (28–32 weeks)	0.7 (1/142)	0.5 (4/831)	0.546	5.9 (4/68)	5.7 (16/281)	1.000
Average birthweight (g)	3427.2 ± 547.6	3318.8 ± 516.7	0.033	2495.6 ± 326.0	2428.6 ± 318.1	0.127
LBW(1500–2500 g)	7.7 (11/142)	16.0 (133/831)	0.010	44.1 (30/68)	59.8 (168/281)	0.019
VLBW (<1500g)	0.0 (0/142)	0.2 (2/831)	1.000	7.4 (5/68)	10.7 (30/281)	0.505
Congenital malformations	0.7 (1/142)	0.4 (3/831)	0.469	0.0 (0/68)	0.7 (2/281)	1.000

OI, ovulation induction; EP, estrogen-progesterone; HDP, hypertensive disorders of pregnancy; GDM, gestational diabetes mellitus; PPROM, preterm premature rupture of membranes; PH, postpartum hemorrhage; PTB, preterm birth; VPTB, very preterm birth; LBW, low birth weight; VLBW, very low birth weight. Data were presented as means ± SD, % (n/N). Differences were analyzed by Student’s t-test, or chi-squared test.

**Table 3** showed obstetrical and neonatal complications after PSM in the two groups. In singleton birth, the prevalence of GDM (7.9% vs. 14.6%; P = 0.037), and LBW (7.9% vs. 14.1%; P = 0.042) was still significantly lower in patients from OI group. However, in twin birth, all complication risks were comparable between patients from two different endometrial preparation protocols.

**Table 3:** Obstetrics and perinatal outcomes of live births in OI and EP groups after propensity-score matching (PSM).

	Singleton			Twin		
	OI	EP	*P*	OI	EP	*P*
Obstetrics outcomes	140	501		64	215	
HDP	3.6 (5/140)	4.0 (20/501)	0.820	6.3 (4/64)	9.3 (20/215)	0.613
GDM	7.9 (11/140)	14.6 (73/501)	0.037	10.9 (7/64)	16.3 (35/215)	0.294
Placenta previa	5.7 (8/140)	6.2 (31/501)	0.836	6.3 (4/64)	8.8 (19/215)	0.614
PPROM	2.9 (4/140)	5.6 (28/501)	0.271	4.7 (3/64)	5.1 (11/215)	1.000
PH	5.7 (8/140)	6.6 (33/501)	0.709	9.4 (6/64)	11.6 (25/215)	0.615
Caesarean section	72.1 (101/140)	75.4 (378/501)	0.426	90.6 (58/64)	87.9 (189/215)	0.549
Perinatal outcomes						
Average gestational age (week)	38.9 ± 2.4	38.5± 2.3	0.247	35.8± 1.8	35.5± 1.7	0.296
PTB (32–37 weeks)	5.7 (8/140)	8.4 (42/501)	0.298	54.7 (35/64)	54.9 (118/215)	0.978
VPTB (28–32 weeks)	0.7 (1/140)	0.4 (2/501)	0.525	4.7 (3/64)	6.5 (14/215)	0.770
Average birthweight (g)	3422.2 ± 541.0	3358.1± 524.7	0.130	2492.6 ± 316.9	2413.4± 320.2	0.257
LBW(1500–2500 g)	7.9 (11/140)	14.4 (72/501)	0.042	50.0 (32/64)	59.5 (128/215)	0.176
VLBW (<1500g)	0.0 (0/140)	0.2 (1/501)	1.000	6.3 (4/64)	11.6 (25/215)	0.252
Congenital malformations	0.7 (1/140)	0.4 (2/501)	0.523	0.0 (0/64)	0.9 (2/215)	1.000

OI, ovulation induction; EP, estrogen-progesterone; HDP, hypertensive disorders of pregnancy; GDM, gestational diabetes mellitus; PPROM, preterm premature rupture of membranes; PH, postpartum hemorrhage; PTB, preterm birth; VPTB, very preterm birth; LBW, low birth weight; VLBW, very low birth weight. Data were presented as means ± SD, % (n/N). Differences were analyzed by Student’s t-test, or chi-squared test.

**Figure 2:**
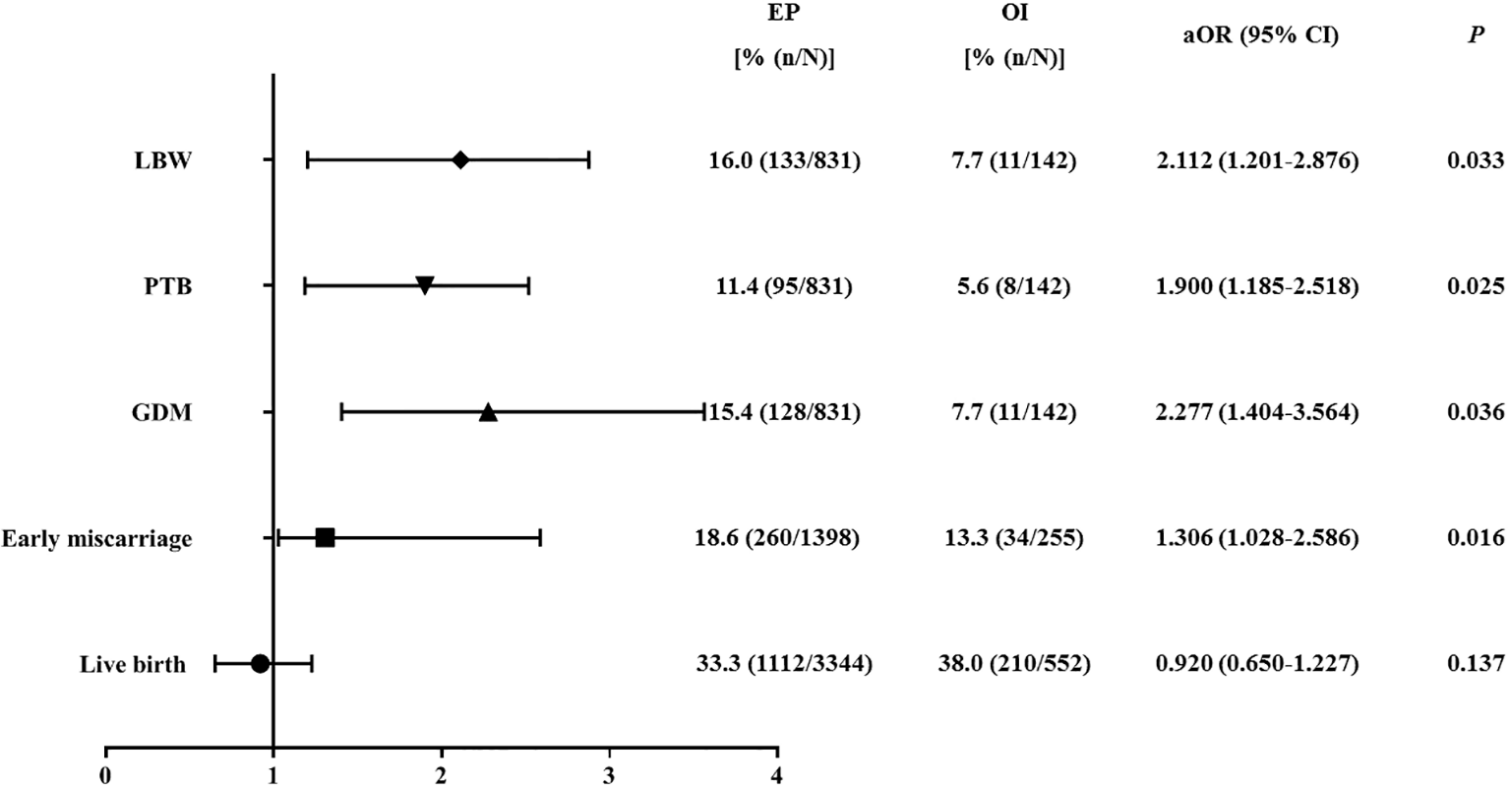
Logistic regression of the pregnancy outcomes, obstetrical/neonatal outcomes in patients with OI versus patients in EP group before PSM. PSM, propensity-score matching; OI, ovulation induction; EP, estrogen-progesterone; aOR, adjusted odds ratio; CI, confidence interval; PTB, preterm birth; LBW, low birthweight; GDM, gestational diabetes mellitus. Adjusted variables included female age, BMI, infertility diagnosis, infertility type, number/stage of embryos transferred, and endometrial thickness.

**Figure 3:**
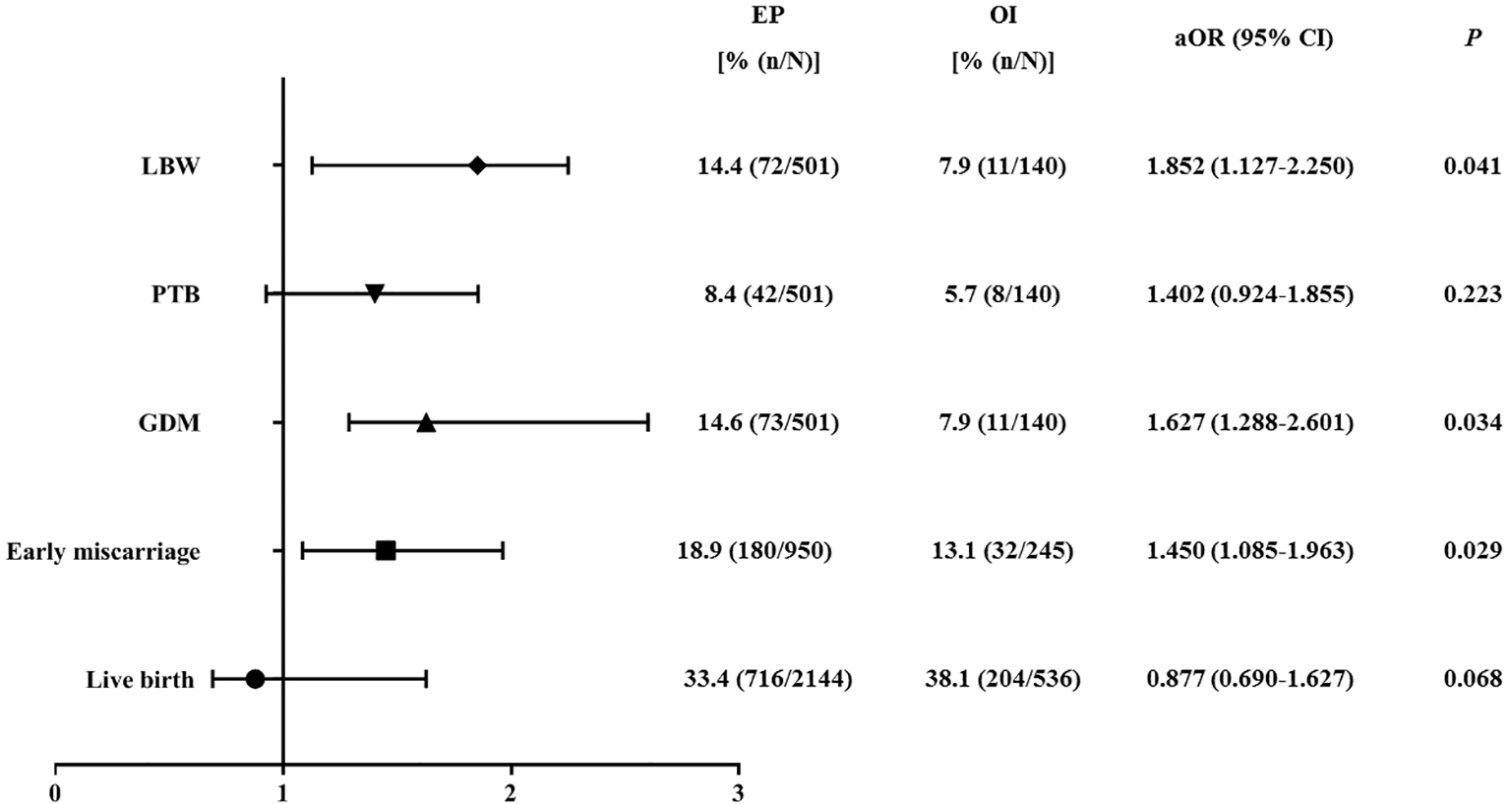
Logistic regression of the pregnancy outcomes, obstetrical/neonatal outcomes in patients with OI versus patients in EP group after PSM. PSM, propensity-score matching; OI, ovulation induction; EP, estrogen-progesterone; aOR, adjusted odds ratio; CI, confidence interval; PTB, preterm birth; LBW, low birthweight; GDM, gestational diabetes mellitus. Adjusted variables included female age, BMI, infertility diagnosis, infertility type, number/stage of embryos transferred, and endometrial thickness.

In order to further explore the association between endometrial preparation protocols and obstetrical/neonatal complications, multivariate logistic regression analysis was performed only in singleton birth before and after PSM. Adjusted covariates were female age, BMI, infertility diagnose, infertility type, number/stage of embryos transferred, as well as endometrial thickness. As shown in **Figure 2**, before PSM, EP resulted in a higher risk of early spontaneous miscarriage rate (OR = 1.306, 95%CI: 1.028-2.586; P = 0.016), GDM (OR = 2.277, 95%CI: 1.404-3.564; P = 0.036), PTB (OR = 1.900, 95%CI: 1.185-2.518; P = 0.025), and LBW (OR = 2.112, 95%CI: 1.201-2.876; P = 0.033). In patients after PSM, as compared with OI, EP still resulted in a higher risk of early spontaneous miscarriage rate (OR = 1.450, 95%CI: 1.085-1.963; P = 0.029), GDM (OR = 1.627, 95%CI: 1.288-2.601; P = 0.034), and LBW (OR = 1.852, 95%CI: 1.127-2.250; P = 0.041) (**Table 3**, **Figure 3**).

## Discussion

Optimal preparation of the endometrium and its synchronization with the embryo to be transferred is imperative to assure the success of frozen embryo transfers. In embryo implantation failure cycles during IVF treatment, two-thirds of them are mainly due to impaired uterine receptivity. With the development of assisted reproductive technology, especially the development of embryo cryopreservation, the quality of fresh and frozen-thawed embryos has been greatly improved. Therefore, how to find a proper protocol to prepare endometrium during FET cycles improving uterine endometrial receptivity has become a critical step of the reproductive process.

Commonly used endometrial preparation protocols include natural cycle, and estrogen/progesterone programmed cycle with or without gonadotrophin-releasing hormone (GnRH) agonist suppression. In addition, another one is ovulation induction cycle using Letrozole. As one of the aromatase inhibitors, Letrozole prevents the conversion of androgens to oestrogens, which releases the hypothalamic–pituitary axis from negative feedback such that a surge in FSH occurs. Letrozole has been used for ovulation induction for more than 20 years. However, little is known about the effect and safety of its use for endometrial preparation in FET cycles.

In 2025, a retrospective cohort study and a randomized controlled trial study in PCOS women both showed that the letrozole ovulation regimen demonstrated comparable clinical pregnancy rates to the programmed regimen in PCOS patients undergoing FET. However, OI protocol requiring only simple luteal support (13, 14). In addition, a systematic review including 8307 women treated with OI protocol and 16,940 women treated with EP protocol showed that Letrozole treatment may offer a modest improvement in reproductive outcomes and a lower risk of some obstetric complications, particularly in women with oligo-anovulation (15). These findings were similar with those from another network Meta-Analysis encompassing 16,082 FET cycles, which showed that the Letrozole ovulation induction protocol presents a lower miscarriage rate than the other endometrial preparation protocols (16). In the current study with larger sample size, results indicate that for patients with ovulatory disorders before PSM, although the endometrial thickness on the day of embryo transfer in patients using OI protocol was thinner, the implantation rate and live birth rate were significantly higher, and the early spontaneous miscarriage rate was significantly reduced as compared with that in EP group. Even after PSM, the live birth rate in the OI group remains significantly higher than that in the EP group, and the early miscarriage rate was notably lower.

Based on results from previous studied, it seems reasonable that ovulation disorder women could be benefit from OI protocol in improving clinical outcomes as compared with EP protocol. However, clinical pregnancy outcome in IVF has been steady in recent years. Currently, both physicians and patients are more focused on the safety of IVF, which mainly include the incidence of obstetrical and neonatal complications. So, compared to EP protocol, is OI protocol still more favorable in reducing obstetrical and neonatal complications? In 2020, Zong et al. performed a retrospective study including 6886 women, and found that EP protocol for endometrial preparation during FET of blastocysts was associated with increased risk of maternal and neonatal complications, as compared to the NC and OI protocol (17). Another similar study also showed that the risks of LGA infants in NC and OI groups were 10.3%, and 7.6%, respectively, which were both significantly lower than that (14.0%) in EP group (18). In addition, another study directly compared the safety between OI protocol and EP protocol. Data also showed that in women with polycystic ovary syndrome undergoing FET, Letrozole use for endometrial preparation was associated with a lower risk of hypertensive disorder of pregnancy than EP protocol for endometrial preparation (9).

So, irrespective of normal ovulation and ovulation disorder women, EP protocol does not have advantage in improving clinical outcomes, and in reducing obstetrical and neonatal complications. And then, what is the reason behind this phenomenon?

For both OI and EP cycles, ovulation occurs, leading to the formation of corpus luteum, which is not present in the EP cycles. The corpus luteum is the main source of progesterone in the luteal phase of the menstrual cycle and the initial two-thirds of the first trimester of pregnancy. Normal luteal function is required for fertility and the maintenance of pregnancy (19, 20). In EP cycles, as compared with OI cycles, luteal function defect is a common issue probably due to low levels of LH and no ovulation, even with luteal phase supplementation. Luteal function insufficient then results in lower pregnancy rates, and significantly higher early miscarriage rates, which is also shown in our data.

In the current study, we also observed an increased risk of GDM and LBW in EP cycles by logistic regression analysis after PSM in singleton births. Even without statistically significance, the incidence of PTB was also higher in EP group as compared with that in OI group. The increased in LBW and PTB could be explained by absence of corpus luteum, which may affect the endometrium and subsequent placental development (21). Aberrant progesterone levels in early pregnancy after EP without corpus luteum may lead to abnormal invasion of the extravillous trophobast, impaired spiral artery remodeling and dysfunction of the trophoblast cells. It has been proven that this altered steroid status is linked to abnormal placentation, PTB, and LBW (6, 22). However, for the association between EP protocol and GDM, it is a little early to put them together, even our data also showed increased GDM in EP group by logistic analysis. The study by Zhang et al. showed that the incidence of GDM after FET in EP group was 13.9%, which was similar with that (12.5%) in Letrozole group (9). Conversely, a large sample size retrospective study from Japan demonstrated a decreased risk of GDM (adjusted odds ratio: 0.52; 95% CI, 0.40-0.68) from pregnancies after EP-FEC in comparison to pregnancies after NC-FET (23). As stated by previous scholars, the discrepancies in findings could be largely attributed to the heterogeneous study population (9). Another point we should not miss is that, according to literature, the use of progesterone during pregnancy as luteal phase support and preterm labor prevention is an important risk factor for GDM in women who conceive following IVF treatment (24). However, another recent study showed that the route of progesterone administration did not significantly affect GDM rates (25). Compared with women in OI group, the dosage of progesterone for endometrial transformation is higher in EP group. Moreover, we observed that patients using EP protocol are more likely to increase dosage of luteal phase supplementation drugs after pregnancy. However, these hypothesis and speculations are from our working experiences, and the mechanism how endometrial preparation protocol affect the risk of GDM needs further exploration.

The strength of this study is single center retrospective cohort study using PSM, which could control potential biases and ensure the reliability of our findings. Moreover, single-center study may magnify the advantages of standard inclusion criteria, treatment and operation protocols. However, some limitations should also be noted. First, due to the retrospective nature of this study, patients were not randomized to EP or OI groups. As mentioned above, the proportion of PCOS women in OI group are relatively higher. After PSM, even though the infertility diagnosis is comparable, the baseline characteristics of two groups could not be ‘true’ similar without using randomization method. Second, not all potential confounders related to clinical outcomes, obstetrical and neonatal complications, such as the history of parity, dietary control, exercise and use of other medicine after pregnancy could be collected and controlled. Third, ovulatory disorder is just a symptom; the underlying causes are complex and varied. Moreover, only clinical outcomes, obstetrical and neonatal complications are compared in this study. The rate of cycle cancellation due to ovulation induction failure is not reported. We should never forget the advantage of EP cycles in programming the thawing and transferring of embryos according to the needs of the IVF laboratory, physician, and most important, the patients (26, 27). Therefore, whether our findings have wilder implication to the general infertile women with FET warrants further prospective, randomized studies in the future.

## Conclusion

In conclusion, the current study suggests that using Letrozole to achieve endometrial preparation prior to FET may yield a decreased risk of early spontaneous miscarriage rate, GDM, and as well as LBW. These results suggest that for ovulation disorder patients undergoing FET, the use of OI protocol with Letrozole could be preferred as compared with EP protocol.

## Data Availability

The original contributions presented in the study are included in the article/supplementary material. Further inquiries can be directed to the corresponding authors.
